# Geriatric assessment for older adults with sickle cell disease: protocol for a prospective cohort pilot study

**DOI:** 10.1186/s40814-020-00673-3

**Published:** 2020-09-17

**Authors:** Charity I. Oyedeji, Katherine Hall, Alison Luciano, Miriam C. Morey, John J. Strouse

**Affiliations:** 1 Department of Medicine, Division of Hematology, Duke University School of Medicine, 315 Trent Dr. Suite 261, DUMC Box 3939, Durham, NC 27710 USA; 2 Duke Claude D. Pepper Older Americans Independence Center, Durham, NC USA; 3 Department of Medicine, Division of Geriatrics, Duke University, Durham, NC USA; 4 Geriatric Research, Education and Clinical Center, Durham Veterans Affairs Healthcare System, Durham, NC USA; 5 Department of Medicine, and Duke Comprehensive Sickle Cell Center, Duke University School of Medicine, Durham, NC USA; 6 Division of Pediatric Hematology-Oncology, Duke University, Durham, NC USA

**Keywords:** Sickle cell, Geriatrics, Aging, Older adults, Geriatric assessment, Functional assessment

## Abstract

**Background:**

The life expectancy for people with sickle cell disease (SCD) has improved tremendously over the last 50 years. This population experiences hemolysis and vaso-occlusion in multiple organs that lead to complications such as cardiopulmonary disease, strokes, and avascular necrosis. These complications can limit mobility and aerobic endurance, similar to limitations that often occur in geriatric populations. These sickle-cell and age-related events lead to frequent hospitalization, which further increases the risk of functional decline. We have few tools to measure functional decline in people with SCD. The purpose of this paper is to describe a protocol to evaluate the feasibility of sickle cell disease geriatric assessment (SCD-GA).

**Methods/design:**

We will enroll 40 adults with SCD (20 age 18–49.99 years and 20 age ≥ 50 years) in a prospective cohort study to assess the feasibility of SCD-GA. The SCD-GA includes validated measures from the oncology geriatric assessment enriched with additional physical and cognitive measures. The SCD-GA will be performed at the first study visit, at 10 to 20 days after hospitalization, and at 12 months (exit visit). With input from a multidisciplinary team of sickle cell specialists, geriatricians, and experts in physical function and physical activity, we selected assessments across 7 domains: functional status (11 measures), comorbid medical conditions (1 measure), psychological state (1 measure), social support (2 measures), weight status (2 measures), cognition (3 measures), and medications (1 measure). We will measure the proportion completing the assessment with feasibility as the primary outcome. Secondary outcomes include the proportion consenting and completing all study visits, duration of the assessment, acceptability, and adverse events.

**Discussion:**

We present the protocol and rationale for selection of the measures included in SCD-GA. We also outline the methods to determine feasibility and subsequently to optimize the SCD-GA in preparation for a larger multicenter validation study of the SCD-GA.

## Background

Sickle cell disease (SCD) is a severe inherited hemoglobinopathy that affects approximately 100,000 people in the USA [1]. Survival has substantially improved over the last 50 years with median survival increasing from 14 years (Diggs et al. [2]), to approximately 40 to 45 years in population-based studies and 61 years in contemporary cohorts recruited at comprehensive programs [2–4]. Individuals with SCD experience a lifetime of sickling and microvascular occlusion that affects every organ of the body [5]. Adults with SCD have frequent hospitalizations. Based on data from the Agency for Healthcare Research and Quality, individuals with SCD have 1.23 hospitalizations per patient per year for individuals age 46–64 and 0.72 hospitalizations per patient per year for individuals age ≥ 65 years [6]. This is substantially more hospitalizations compared to the general non-SCD population, which is 0.11 hospitalizations per patient per year for individuals age 45–64 and 0.35 per patient per year for individuals age ≥ 65 [7].

Aging is defined as a deterioration in physiological function that occurs over time [8]. Individuals with similar chronological age often vary in their functional age [9]. Adults with SCD demonstrate substantial and early deterioration of multiple organ systems that leads to complications seen frequently in geriatric populations, such as cardiopulmonary disease, sensory deficits, and a decline in physical and cognitive function [10–12]. They are particularly at increased risk of lower extremity functional decline due to complications such as avascular necrosis of the hips often requiring surgical intervention [13].

SCD providers often use SCD complications, chronological age, laboratory data, healthcare utilization, and subjective measures of performance status to assess overall health and risk of adverse outcomes. In one of the few studies with multiple direct measures of physical function, adults with SCD had lower pulmonary function, grip strength, and functional capacity measured by six-minute walk test (6MWT) compared to predicted norms for age and gender [14]. The 6MWT is the only physical functional assessment routinely used to assess patients with SCD. The Adult Sickle Cell Quality of Life Measurement System, ASCQ-Me℠, and Patient-Reported Outcome Measurement Information System, PROMIS®, are subjective measures used to assess health and function in individuals with SCD [15]. These measures are useful in that they do provide patient-reported data about important areas of health in adults with SCD such as sleep, pain, fatigue, and mental health. However, as individuals with SCD age, the current condition-specific assessment tools available are limited in their ability to capture both the SCD and age-related areas, such as objective measures of physical function, frailty, dependency, and polypharmacy.

There is a need for comprehensive and objective measures of health and function that are tailored to the specific needs of older adults with SCD. These measures should be simple and brief enough to be easily integrated into a clinical setting. When selecting measures to include in an assessment, SCD-specific factors, such as acute and chronic pain, avascular necrosis (AVN) of the joints, early onset of silent and overt strokes, pulmonary hypertension, and cultural differences, require careful consideration. Assessment tools used for non-SCD older populations, like geriatric assessment, are useful in assessing age-related conditions and functional decline; however, these measures have not been validated in adults with SCD. There is a need for validated assessment tools to evaluate aging adults with SCD to properly identify and address the unique issues in this population.

Clinicians have used geriatric assessment to identify the capabilities and vulnerabilities of older individuals by measuring physical function, cognitive function, psychological state, nutritional status, social support, comorbidities, and medications [16]. Geriatricians routinely use geriatric assessment in clinical practice. Oncologists have also widely used geriatric assessment to assess risk of toxicity and mortality related to chemotherapy. Interventions based on geriatric assessment can improve survival, preserve independence, and restore mobility [17, 18].

Our goal is to develop the first geriatric assessment for older adults with sickle cell disease (SCD-GA). Our rationale for developing SCD-GA is to create a standard method for assessing risk of adverse outcomes, address the unique needs of this growing population of older adults with SCD, and identify modifiable deficits to guide the development of interventions to improve function and quality of life. We hope these interventions will also reduce frailty and mortality.

### Study aims and objectives

This study aims to assess the feasibility of SCD-GA, by the proportion completing the assessment and consent, duration of the assessment, acceptability, and adverse events. Our aim is also to assess the feasibility of repeat measures at different time points by performing the SCD-GA at steady state, post-hospitalization, and 12 months after the initial assessment. We will use the results of this pilot study to select measures to be included in the revised SCD-GA and to optimize the design of a multicenter validation study of this revised assessment. We also will develop interventions based on deficits identified by the SCD-GA.

## Methods/design

### Study design

This study is a prospective cohort pilot study designed to assess the feasibility of a geriatric assessment for SCD. It includes a combination of patient-reported questionnaires and provider-administered physical and cognitive assessments. The study has been approved by the Duke Institutional Review Board (protocol number: Pro00100358; version 1.11; IRB reference date: July 18, 2019). All participants will provide written informed consent prior to initiation.

### Study population and setting

There are approximately 650 active adults in our comprehensive sickle cell center located in an academic medical center in the Southeastern United States. Thirteen percent of the adults with SCD are aged 50 years or older. This study will include adults with SCD at a single comprehensive sickle cell center with planned enrollment of 40 participants (twenty aged 18–49.99 and twenty aged ≥ 50 years). We defined older adults as aged ≥ 50 years based on the life expectancy for SCD [4]. Since this is a pilot study and the primary outcome is feasibility, we did not use power calculations to determine sample size, and the study is not powered to predict clinical outcomes. The justification for the sample size is based on the number and frequency of clinic visits for older adults in the sickle cell center and the preference for participants with varying SCD genotypes and complications to provide a wide range of feedback on acceptability and feasibility of performing the SCD-GA [19].

#### Eligibility criteria

We will include participants that (1) have a diagnosis of SCD confirmed by hemoglobin electrophoresis, high-performance liquid chromatography (HPLC), or genotyping; (2) age greater than or equal to 18 years of age; and (3) speak fluent English. We will exclude patients if they (1) have been previously diagnosed with moderate to severe cognitive impairment by their usual provider, (2) are unable to self-consent, or (3) are wheelchair-bound.

### Data collection

We will record demographics, complications from SCD, comorbidities, social history (e.g., education, household income, employment status, insurance), and steady-state lab values. We will collect study data and manage the data using REDCap electronic data capture tools hosted at Duke University [20, 21]. We will include consensus measures such as demographics, social history, and SCD complications from the Phenotypes and eXposures (PhenX) Toolkit when available [22]. We will collect data on last hospitalizations, emergency department visits, and day hospital visits by reviewing the medical record and verbally confirming with participants to ensure they are at steady state at the time of their baseline SCD-GA.

SCD providers, geriatricians, and exercise specialists from the Center for Aging and Human Development collaboratively selected the measure included in the SCD-GA. We included assessment tools validated in the oncology geriatric assessment and supplemented them with additional physical and cognitive measures [16, 23]. The measures in the oncology geriatric assessment were originally selected based on validity, brevity, reliability, and prognostic value [16]. For the SCD-GA, we added 5 additional physical functional assessments to gain a better understanding of which measures would be most feasible in this population. The SCD investigators attended a physical function assessment in older adults workshop to learn about commonly used measures. Investigators who were SCD physicians received guidance on how to properly perform and analyze physical function assessments and had ongoing training throughout the study to ensure proper and consistent technique.

### Timing of assessments

All participants will receive a baseline SCD-GA at steady state, which is defined as greater than 6 weeks after their last hospitalization and greater than 2 weeks after their last emergency department or SCD day hospital visit. If participants are hospitalized during the study period, they will receive one additional assessment 10–20 days after the hospitalization. If multiple hospitalizations occur during the 12-month period, the participant will not receive additional post-hospital assessments. Each participant will receive an assessment at least 12 months after the first assessment, which will also be at steady state.

### Outcome measures

Feasibility studies play an integral role in improving the quality of research by allowing the investigator to address flaws in the methodological design before conducting a large-scale study [24]. The endpoints of this study are based on Consolidated Standards of Reporting Trials (CONSORT) extension to pilot and feasibility trials guidelines [24, 25]. We will determine feasibility by the proportion completing the initial SCD-GA as the primary outcome. Secondary feasibility outcomes include proportion completing consent and all study visits, duration of the assessment, and adverse events. We will assess acceptability by a satisfaction survey that participants will complete at the end of each study visit.

#### Proportion completing SCD-GA

We defined the proportion completing the SCD-GA as the proportion of participants consented that complete the SCD-GA. We will further characterize this as the proportion of participants who present for all study visits, complete the self-reported questionnaires, and complete all physical assessments. We will calculate the proportion completing the SCD-GA for the baseline visit, post-hospital visit (for those who are hospitalized during the study), and at end of study assessment. An acceptable cutoff for proportion of completion will be ≥ 80%.

#### Proportion consenting

We defined the proportion consenting as the proportion of patients signing a consent form out of those who were approached to participate. We will record the reason individuals decide not to participate in the study. An acceptable cutoff for proportion consenting will be ≥ 80%.

#### Retention

We defined retention as the proportion of participants who remain in the study and complete the 12-month follow-up visit. The denominator is the total number of participants that are enrolled and complete their baseline assessment. An acceptable cutoff for retention will be ≥ 80%.

#### Duration of the assessment

We will record the start and end times for the SCD-GA starting with the recording of demographic data for the initial visit and starting with social history for all subsequent visits. We will not record the duration of physical assessments separately. We will intersperse physical function assessments between patient-reported questionnaires and scheduled breaks to permit sufficient time for recovery between physical measures. Participants will complete demographic data and the reading test at the initial visit. An acceptable duration of the assessment will be ≤ 120 min and at least 80% reporting the length as appropriate.

#### Adverse events

Investigators performing the assessments will record adverse events. We will clarify if adverse events are related or unrelated to the SCD-GA. Investigators will send a report to the designated Data and Safety Monitoring Officer every 6 months. The Data and Safety Monitoring Officer is a hematologist familiar with but not involved in the study. Acceptability cutoff for adverse events is no moderate or major adverse events.

#### Acceptability

We will determine acceptability of the SCD-GA by a satisfaction survey at the end of each study visit. We will include questions about satisfaction with the time it takes to complete the entire assessment, whether there are questions that are difficult to understand, uncomfortable, or upsetting, and suggestions about measures that should be added or removed. We will ask participants to elaborate on reasons a particular measure is not acceptable and provide open-ended feedback on suggestions for improving the SCD-GA as a whole. Acceptability cutoff will be < 20% reporting difficulties in understanding measures or reporting questions as upsetting or uncomfortable.

### Enrollment and retention strategies

Study participants will consist of a convenience sample of patients recruited from a single SCD center. We will recruit participants from the investigators’ clinic patient panel. Investigators will also partner with other providers in the SCD center for enrollment. SCD providers and clinic nurses will notify investigators when patients meet study criteria. We will approach participants during routine clinic visits or while in the SCD day hospital. Prior to enrollment, we will screen each participant for inclusion and exclusion criteria. Each participant will provide written informed consent prior to initiation of assessments. For retention, we will leverage the electronic medical record to track hospitalizations and scheduled clinic visits during the study period. The inpatient SCD service will also notify investigators when participants are hospitalized and when they are returning to clinic for a post-hospital follow-up.

### SCD-GA measures and rationale for selection

The following are the measures selected for the SCD-GA (Table 1). This assessment focuses on key areas at the cross-section of SCD and geriatrics. We found that it is best to use a multidisciplinary approach when selecting measures for the SCD-GA [26]. The investigators are adult SCD clinicians collaborating with exercise experts and geriatricians from a Pepper Older Americans Independence Center (OAIC). This collaboration between SCD and geriatric specialists is a novel concept. SCD has historically been a disease of children and young adults until modern-day initiatives, such as penicillin prophylaxis and hydroxyurea, improved survival with some individuals now even becoming octogenarians [27, 28].

**Table 1:** Focused geriatric assessment domains and measurements

Domains	Assessment tools in each domain
Functional status	▪ ADL and IADL (subscales of the OARS) ▪ MOS Physical Functioning Scale ▪ Karnofsky Performance Status (KPS)—self and physician ▪ Number of falls in last 6 months ▪ Timed Up and Go (TUG) ▪ Usual gait speed ▪ Dual-task performance ▪ Six-minute walk test ▪ Grip strength ▪ 30-second chair stand
Comorbid medical conditions	▪ Patient-reported comorbidity checklist and chart extraction
Psychological state	▪ Mental Health Inventory-18
Social support	▪ MOS Social Support Survey ▪ MOS social activities
Nutritional status	▪ Body mass index ▪ Unintentional weight loss
Cognition	▪ The Blessed Orientation-Memory-Concentration Test ▪ Montreal Cognitive Assessment (MoCA) ▪ Wide Range Achievement Test-5 (WRAT-5)
Medications	▪ Comprehensive list of medications

We included all measures that were previously validated in the oncology geriatric assessment and added additional physical and cognitive functional measures. The measures in the SCD-GA differ from the oncology geriatric assessment in that the oncology geriatric assessment includes the Timed Up and Go (TUG) and the Blessed Orientation-Memory-Concentration (BOMC) Test as the only physical and cognitive measures [16]. For the SCD-GA, we included additional physical and cognitive functional measures to evaluate the early physical and cognitive decline that occurs in the SCD population [14, 29]. We also included several SCD-specific questions on healthcare utilization, SCD complications, and pain.

There are 7 domains total: functional status (5 surveys and 6 physical assessments), comorbid medical conditions (1 patient checklist), psychological state (1 mental health measure), social support (2 surveys), weight status (body mass index and patient-reported weight loss), cognition (2 assessments), and medications (patient-reported list). The additional physical functional assessments include usual gait speed, 6MWT with heart rate recovery, seated grip strength, 30-second chair stand, and dual-task performance. We also added the Montreal Cognitive Assessment (MoCA) as an additional cognitive measure and a subtest of the Wide Range Achievement Test 5th Edition (WRAT-5) as a reading skills test to account for differences in academic achievement beyond stated education level.

### Functional status

We will assess physical function using a combination of previously validated self-administered surveys and provider-administered physical function assessments [16, 23]. We will compare the results of each physical function assessment to normative values based on age and gender where such data are available [30].

#### Activities of Daily Living and Instrumental Activities of Daily Living

Activities of Daily Living (ADL) and Instrumental Activities of Daily Living (IADL) are subscales of the Older American Resources and Services Multidimensional Functional Assessment Questionnaire (OMFAQ) developed to assess the extent to which elderly individuals are able to function independently and to assess service utilization [31]. The responses are on a 3-point Likert scale ranging from “without help” (2 points) to “completely unable” (0 points). This is of particular interest in the older SCD population since there is no data regarding their level of dependence and types of services required as they age, such as the types of home safety modifications to implement and the need for skilled nursing facilities.

#### MOS Physical Functioning Scale

The Physical Functioning Scale is a component of the Medical Outcome Study (MOS) 36-item Short Form (SF-36), which is a compilation of patient self-reported quality of life measures used to monitor healthcare outcomes in well and chronically ill adults [32]. The Physical Functioning Scale is a 10-item 3-point Likert scale with a higher score indicating better physical function. It is an appropriate measure for adults with SCD, who are assumed to be more independent than typical geriatric populations, since it goes beyond limitations in daily self-care.

#### Number of falls

Falls pose a substantial threat to the independence of older adults and are associated with risk factors such as sedative use, acute illness, cognitive impairment, and environmental hazards [33]. Participants will report the number of falls in the last 6 months at each visit. Many older adults with SCD are treated with opioids, antidepressants, and other medications that historically have increased the risk of falls in the elderly [34].

#### Karnofsky Performance Status

Karnofsky Performance Status (KPS) is a subjective measure of an individual’s global physical ability [35]. Responses are on an 11-point scale that correlates with a percentage that increases by increments of 10 ranging from “dead” (0%) to “normal, no evidence of disease” (100%). In this study, we will collect both provider- and patient-reported KPS. The KPS is not well studied in patients with SCD.

#### Usual gait speed

Usual gait speed is a widely used physical performance functional measure that alone can predict functional decline and mortality. Participants will walk at their usual pace on a 3-m (10 ft) course with a 1-m acceleration zone before and a 1-m deceleration zone after the 3-m walking course for 2 trials. We will use the fastest speed for the analysis. There is a large body of data that has established the reliability and validity of usual gait speed as a measure of physical function and as a predictor of healthcare utilization and mortality [36, 37].

#### Timed Up and Go

The TUG test assesses physical mobility and measures the time it takes to rise from a standard height chair (46 cm), walk a distance of 10 ft (3 m), turn, walk back to the chair, and sit down again. It is often included as a component of physical assessments because it requires no special equipment. It is the only provider-administered physical function assessment in the oncology geriatric assessment [16]. A TUG test > 12 s indicates an increased risk of falls.

#### Six-minute walk test

The 6MWT is a test of aerobic endurance that has been well validated in both geriatric and SCD populations; however, there are no data in older adults with SCD [14, 38, 39]. It is used in screening for pulmonary hypertension, a complication more common in older adults with SCD and is associated with increased mortality [10, 40]. During the 6MWT, we will instruct the participant to “cover as much ground as possible” by walking for 6 min on a 20-m walking course up-and-back and around 2 cones at each end of a quiet hallway in adult sickle cell clinic. We will record both 6-min and 2-min walking distances [30]. We also will record heart rate recovery at 1 and 2 min. Children with sickle cell anemia have poor heart rate recovery [41]. Attenuated heart rate recovery is associated with an increased risk of cardiovascular events and all-cause mortality in the general population [42].

#### Seated grip strength

Seated grip strength is a measure of upper body strength and is highly correlated with mobility, physical activity, and quality of life [43]. We will use the Jamar Technologies Hydraulic Hand Dynamometer to measure grip strength in triplicate for both hands while the participant remains seated in a standard height chair (46 cm) with feet flat on the floor and elbow snug against the body. We will compare the maximal strength (in kilograms of force) of the dominant hand, determined by the dynamometer, to normative values based on age and gender [44]. Individuals in the general population with lower grip strength have an increased risk of all-cause mortality and mortality from cardiovascular and respiratory disease [45]. A study of young adults with SCD showed that grip strength correlated with pulmonary function [14].

#### 30-second chair stand test

The 30-second chair stand test is a measure of lower body strength. Participants will perform the 30-second chair stand test by first sitting in the middle of a standard height chair (46 cm) without arms. Investigators will instruct the participant to stand up and sit back down with arms across their chest as many times as possible in 30 s. We will compare results to normative values based on age and sex [46]. The 30-second chair stand test has been validated in various elderly populations and is a marker of functional independence [47]; however, there is no data on how it performs in individuals with SCD. Older adults with SCD are at increased risk of lower extremity functional decline due to complications such as avascular necrosis of the hips [13].

#### Dual-task performance

Dual-task performance assesses the effects on mobility of simultaneously performing two tasks, which is typically a cognitive and motor task. For this test, we will ask the participants to walk at their usual gait speed for 1 min and perform a verbal fluency task (generate as many words beginning with a single letter in 1 min). F, A, and S are the most commonly used letters for verbal fluency and are all classified as “easy” letters [48]. Participants will do each task once individually, then simultaneously twice using a different letter on each attempt. We will calculate the dual-task effect by assessing the relative change in performance during single- and dual-tasking. We will plot the results as a percentage to determine if there is cognitive or motor interference or facilitation [49]. The utility of this test in individuals with stroke [50] is of particular interest since nearly 40% of individuals with sickle cell anemia have silent strokes by age 14 and 24% have an overt stroke by age 45 [51–53].

### Comorbid medical conditions

As individuals get older, the number of comorbid medical conditions increases. Comorbidities such as cardiovascular disease, cerebrovascular disease, cancer, and diabetes are predictive of mortality [54]. We will record comorbidities using the OARS Physical Health questionnaire. Participants will select comorbidities from a list of conditions and the degree to which the condition interferes with their daily activities on a 3-point scale of “not at all” to “a great deal.” They will also select from a list of SCD complications. Individuals with SCD are at increased risk for retinopathy and sensorineural hearing loss so they will also rate their vision and hearing [55, 56].

#### Pain

Pain has a significant impact on multiple domains of health for adults with SCD. To assess the impact pain has on the various measure in the SCD-GA, we will collect data on healthcare utilization for pain at each study visit. We later revised the protocol to include patient-reported outcomes for pain using the PROMIS® Pain Interference and PROMIS® Pain Severity, which patients will complete during the post-hospital follow-up and 12-month follow-up assessments.

### Psychological state

Mental health is important in every population and is a common complication for individuals with a chronic illness. We will assess psychological state using the Mental Health Inventory (MHI-18), which has 4 subscales that include anxiety, depression, behavioral control, and positive affect. A total score is derived from all items and for each subscale ranging from 0 to 100, with higher scores indicating better mental health [57, 58]. A third of adults with SCD have depression, which is associated with worse healthcare utilization and health-related quality of life [59, 60]. Silent strokes, a complication of SCD that also occurs in non-SCD geriatric populations, are also associated with depression in the elderly [52, 61]. There is no data on mental health in older adults with SCD.

### MOS Social Support

Social support is an integral part of a geriatric assessment. Individuals with low quantity and quality of social relationships have an increased risk of mortality and morbidity [62, 63]. The MOS Social Support instrument is a self-administered questionnaire on an individual’s perceived availability of social support [64]. It includes 18 items that address 4 dimensions of social support (emotional/informational, tangible, affectionate, and positive social interaction) on a 5-point Likert scale ranging from “none of the time” (1 point) to “all of the time” (5 points). An overall social support index is calculated using the mean of all items and converted to a 100-point scale.

### MOS Social Functioning

The Social Functioning subscale is a component of the MOS. It includes 3 items that measure the extent that an individual’s physical health interferes with their social activities. It addresses the amount of time physical health interferes with social activity, change in social activities over time, and social activity limitations compared to an individual’s peers [32].

### Weight status

We will assess body mass index (BMI) and screen for unintentional weight loss in the last 6 months. BMI is calculated by dividing the weight in kilograms by height in meters squared. In the elderly, both high and low extremes of weight and a weight loss of 5% or more are associated with an increased risk of mortality [65, 66]. Individuals with SCD have historically had low body weight; however, BMIs have recently been rising, which may increase the risk of obesity-related diseases [67, 68].

### Cognition

#### Blessed Orientation-Memory-Concentration Test

The BOMC Test is a validated 6-item questionnaire to screen for cognitive deficits [69]. It measures temporal orientation, short-term memory, and concentration. The BOMC performs similarly to the Mini-Mental Status Exam; however, some prefer the BOMC for its rapid and completely verbal administration. A score of greater than 9 is a sensitive screen for cognitive impairment [70].

#### Montreal Cognitive Assessment

The MoCA is a performance-based cognitive assessment tool that measures the following domains: visuospatial skills, executive functions, memory, attention, calculation, concentration, language, abstraction, and orientation. The cutoff established for mild cognitive impairment is a score of less than 26/30. The MoCA has a sensitivity and specificity superior to the Mini-Mental Status Exam [71]. In a study of 1419 community-dwelling African Americans, 80% met criteria for mild cognitive impairment with most participants missing the same items (cube drawing, delayed recall, sentence repetition, and abstraction) [72]. This suggests that the established cutoff of 26 may not be appropriate for African American populations. In a cross-sectional study of 100 adults with SCD, 46% of participants scored < 26 [29].

#### Word Reading Subset of Wide Range Achievement Test

Word Reading Subtest of the WRAT-5 measures literacy and reading grade equivalent [73]. We will ask participants to read a series of words aloud slowly and clearly. We will convert raw scores to a reading grade equivalent based on age.

### Medications

Polypharmacy, defined as being on more than 5 prescribed medications, increases the risk of drug-drug interaction and adverse drug events, especially in the elderly [74]. We will ask participants to record their medications. Participants will also record their use of short-acting and long-acting opioids. We will compare their reported medications to their medication list in the electronic medical record. As individuals with SCD age, they continue to have pain episodes that are treated with opioids and adjunctive medications [10]. There is no data on the appropriate age for adults with SCD to start minimizing potentially inappropriate medications based on the American Geriatric Society (AGS) Beers Criteria [75].

### Statistical analysis

We will evaluate the feasibility of SCD-GA by computing the feasibility outcome rates, as defined above, overall and by age group using binomial exact methods to calculate point estimates and 95% CI. Based on a proportion of 50%, we will estimate the proportion of assessments completed and the proportion of participants and individual measures with missing data with a 95% confidence interval of ± 0.16. We will describe satisfaction survey responses using simple descriptive statistics and qualitative review for recurring themes.

Given that the primary purpose of this study is to evaluate feasibility and acceptability, the remaining analyses will be exploratory and descriptive in nature. We will use descriptive statistics and visual displays to summarize the demographic data and unadjusted results of the SCD-GA measures. We will identify tools with low variability in responses by evaluating maxima and minima for floor and ceiling effects. In addition, we will use correlation matrices to assess for redundancy in the collected data. We will model the bivariate relationships between age and performance on the proposed SCD-GA component measures by linear regression and compare the older (age ≥ 50 years) and younger (age 18–49.99) participants and study participants to age- and sex-matched normative data. Outcomes for statistical models will be computed separately for (1) baseline and (2) approximate 12-month follow-up with baseline value of the outcome as a covariate. The modeling strategy will include age and gender as a covariate in adjusted models. As in most feasibility pilot studies, results will not be interpreted as definitive in size or direction, or causal in their effect. We will conduct analyses in R Statistical Software version 3.6.1 (Foundation for Statistical Computing, Vienna, Austria) and Stata 16 software (StataCorp. 2019. Stata Statistical Software: Release 16. College Station, TX: StataCorp LLC).

### Optimization plan

After completion of the study, we will revise the SCD-GA to create a focused geriatric assessment. Comparing SCD-GA baseline results in adults across a wide age range will inform clinicians as to the appropriate age to initiate the SCD-GA. We will determine the feasibility and acceptability of repeated assessments and the sensitivity of these measures to change by assessing post-hospitalization follow-up, 12-month follow-up, and change from baseline compared by age group. We will eliminate measures that perform poorly (difficult to complete, limited variability), and modify the assessment to enhance usability and cultural appropriateness. We will keep measures that provide adequate representation from each domain. We will also prioritize measures that identify modifiable risk factors and intervenable areas. We will keep measures previously validated in the SCD literature, such as the 6MWT, and measures validated in the geriatric literature that predict important functional outcomes. We will use analytic results to limit redundancy and avoid floor and ceiling effects. We will also prioritize keeping tools that are shorter in length, time, and lower in complexity. Complexity will be determined by the amount of equipment and training required to administer the tool. We will prioritize keeping measures that take no more than two 1-h sessions for a provider or research assistant to learn how to administer. We will also eliminate tools that are high in burden on providers and/or patients based on results of satisfaction survey and experience of investigators administering the assessment. We will remove measures if greater than 25% of subjects failed to answer at least one item or if greater than 20% report that the measure is upsetting or difficult to understand. The goal after optimization will be to have an SCD-GA that has a median time to completion less than 45 min.

## Discussion

**Fig. 1:**
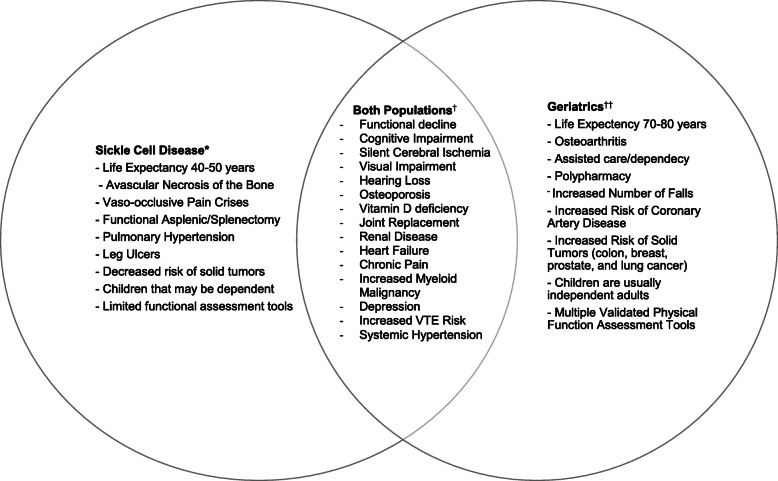
Comparison of characteristics and complications of sickle cell disease vs. geriatrics. *Individuals with SCD have a shorter life expectancy [3, 4], avascular necrosis of the bone [76], vaso-occlusive pain crises [10, 77], asplenic/splenectomy [10], increased pulmonary hypertension [78], leg ulcer [10], lower risk of solid tumors [79], more likely to have younger children that are still dependents, and there are few validated functional assessment tools for this population [80]. ^†^Both geriatric populations and individuals with SCD have functional decline/disability, cognitive impairment [29, 61], silent cerebral ischemia [52, 61], vision loss [81, 82], hearing loss [11, 82], osteoporosis [76, 83], vitamin D deficiency [76, 83], joint replacement [10], renal disease [10, 84], heart failure [77, 78], chronic pain [10, 85], a higher risk of myeloid malignancies compared to the general population [79, 86], high rates of depression [59, 87], and increased VTE risk [88, 89]. ^††^Geriatric populations’ life expectancy 70–80 years [90], osteoarthritis [91], institutionalization, polypharmacy [74], experience falls [33], coronary artery disease [77], increased risk of solid tumor [92], have older children (non-dependents), and multiple functional assessment tools [26]

There is a need for appropriate assessment tools and interventions that will improve function, quality of life, and mortality for adults living with SCD. As knowledge and treatment options for individuals with SCD improve, the life expectancy will likely continue to improve. Aging with SCD has not previously been a widely studied area given the historically shorter life expectancy for individuals with SCD relative to the general population. Older adults with SCD are unique in that they are faced with issues at the intersection of geriatrics and SCD. Many complications overlap, such as cognitive impairment, strokes, increased venous thromboembolism risk, depression, and vision loss (Fig. 1). However, they still face SCD-specific complications, such as recurrent vaso-occlusive pain crises, avascular necrosis, and pulmonary hypertension that sometimes worsen with age (Fig. 1). Addressing both their age- and SCD-related issues requires specific knowledge. In addition, many of these complications occur earlier in life due to premature functional aging caused by repetitive vaso-occlusion in every organ [10]. We need longitudinal data to determine the appropriate age to initiate geriatric assessments in adults with SCD. This will permit early detection of functional decline and optimal timing of interventions to address deficits identified by the SCD-GA.

Identifying and addressing functional deficits may improve the longevity and quality of life for people with SCD. For many individuals, longevity becomes less important if the quality of life is poor. Multiple studies have shown that interventions based on the geriatric assessment improve not only survival for older adults in the general population, but also patient-important outcomes such as mobility, endurance, and strength [93, 94]. Geriatric assessment has been shown to preserve independence when implemented in hospitalized older adults [95]. This is of interest for older adults with SCD who are frequently hospitalized and often become frail after a prolonged hospitalization.

The SCD-GA may be of particular benefit to individuals with SCD receiving new pharmacologic therapies and curative options that require conditioning with chemotherapy. The geriatric assessment for oncology has been shown to improve chemotherapy tolerance and reduce toxicity [96]. The outcomes of the SCD-GA can be used to assess the risk of poor outcomes in response to therapies and can serve as useful clinical endpoints to demonstrate how new SCD therapies impact function.

When analyzing the results of the SCD-GA, we will need to give careful consideration to ethnic and racial differences in the outcomes. The majority of individuals with SCD living in the USA are of African descent. Many of these measures used in the geriatric assessment have not been validated in African Americans, which limits generalizability. This is due to a disparity in inclusion of black people in functional assessment research studies. This disparity has been mainly due to systemic racism that has promoted a system of predominantly white clinicians and scientific investigators, many of whom carry implicit biases in favor of white patients [97]. In addition, many black people have not participated in these studies due to mistrust in the healthcare system because of historical and experienced mistreatment and discrimination [98].

Discrimination not only affects recruitment of black people in research studies, but directly affects the quality of care and many of the health measures we have included in the SCD-GA, such as social functioning and psychological state [99, 100]. Moreover, the stigma associated with having SCD and being on chronic opioids has a direct impact on health outcomes [99]. In future studies of the SCD-GA, we will assess how stigma impacts health outcomes in older adults with SCD using the validated Measure of Sickle Cell Stigma (MoSCS) instrument [99]. Patients with SCD also experience acute and chronic pain that not only leads to stigmatization in the community and healthcare settings, but also influences their physical and mental health. In this study, we will be collecting information on socioeconomic status to assess how these social determinants of health impact the feasibility and results of the SCD-GA.

There also may be cultural differences in the language used in the questionnaires that can affect the validity of the results. The MoCA is of particular concern since it was originally developed and validated in the French-speaking city of Montreal that has a demographic very different from the Southern United States. Previous studies have shown that African Americans have lower MoCA scores compared to US whites. Quality of education has been shown to impact the results, and the cultural appropriateness of the measure remains unclear. In addition, when analyzing the results of the ADLs and IADLs, it may not be adequate to simply compare results of older adults with SCD to normative values, which are based on a predominantly white US population. One will have to take into account that community-dwelling older African Americans have higher rates of self-reported disability compared to older whites [101]. Older African Americans in the general population also have lower physical performance scores, such as slower gait speed, compared to older whites [101, 102]. Previous geriatric assessment studies have not appropriately addressed these racial disparities in function and health outcomes. For this study, we will use previous functional assessment studies that include African American participants as normative values for comparison when available. Validation studies will be required to determine the appropriate cutpoints for adults with SCD at different ages.

There are many challenges to implementing a geriatric assessment for adults with SCD. Individuals with SCD experience cardiopulmonary complications, excruciating pain, and decreased mobility secondary to avascular necrosis of the joints. Many of these complications increase with age [10]. These complications may limit their ability to complete portions of the physical assessment, especially during or after hospitalization. Pain may also confound the results of measures such as physical function, physical performance tests, and psychological state. In this study, we are collecting data on pain and opioid utilization to assess the degree this affects the results. Secondly, clinics may have limited time and infrastructure to implement the SCD-GA. A multidisciplinary approach is optimal. After validation of a streamlined SCD-GA, we will need to assess the feasibility of implementation of the assessment into a variety of outpatient clinic settings. Finally, there are challenges to implementing interventions to address the deficits identified by geriatric assessments. Interventions for impaired physical function have the most evidence and are the easiest to measure outcomes. However, interventions have to be individualized to the disability of the participant. For some participants, the geriatric assessment may serve as an intervention by making the individual aware of deficits, thus promoting lifestyle changes.

### Future directions

After completion of the study and optimization of the SCD-GA, we plan to perform a larger multi-institutional study to determine validity and describe trajectories of function. We will assess the content and predictive validity to ensure measures are truly representative of all 7 domains and determine if the measures can predict patient-important outcomes such as health-related quality of life, mobility, hospitalizations, and mortality. We will subsequently develop an exercise intervention to improve physical function based on deficits identified by the SCD-GA.

## Data Availability

The datasets used and analyzed during the current study are available from the corresponding author on reasonable request.

## Abbreviations

SCD: Sickle cell disease
SCD-GA: Sickle cell disease geriatric assessment
6MWT: Six-minute walk test
AVN: Avascular necrosis
TUG: Timed Up and Go
BOMC: Blessed Orientation-Memory-Concentration
OAIC: Older Americans Independence Center
HPLC: High-performance liquid chromatography
PhenX: Phenotypes and eXposures
CONSORT: Consolidated Standards of Reporting Trials
MoCA: Montreal Cognitive Assessment
WRAT-5: Wide Range Achievement Test 5th Edition
ADL: Activities of Daily Living
IADL: Instrumental Activities of Daily Living
OAFQ: Older American Resources and Services Multidimensional Functional Assessment Questionnaire
MOS: Medical Outcome Study
SF-36: 36-item Short Form
KPS: Karnofsky Performance Status
MHI-18: 18-item Mental Health Inventory
BMI: Body mass index
AGS: American Geriatric Society
MoSCS: Measure of Sickle Cell Stigma
